# Observations on Hydrophobia

**Published:** 1808-12

**Authors:** 


### *Observations on Hydrophobia, by* Dr. Bradley.

It is not presumed that these observations will suggest any thing new or interesting to those medical practitioners, who have availed themselves of the usual means of acquiring information. They are designed for those who have not been so fortunate; or rather for the general public. Whenever we discover errors, whether arising from want of information, or *false* opinions, which lead to dangerous or calamitous consequences, no apology can be necessary for attempting to dissipate them.

An error obtains very generally in the metropolis, that the frequent cry of "mad dog" does not arise from any real disease existing in that animal, but from the avaricious

---

cious desire of purchasing its skin cheaply. If this error had been exclusively confined to the lower orders, I should not have obtruded myself upon the attention of the public at this time; but I state with concern, that many practitioners of medicine in London do not believe in the existence of any such disease as that commonly called *Hydrophobia*, or more properly *rabid erethism*. This error has led to fatal consequences within my own observation; and I therefore trust that the following suggestions will not be deemed irrelevant.

Whenever any person has been bitten by a dog or cat, the animal should be immediately confined, and two or three days will shew whether there is any cause for preventive measures. If rabid symptoms appear in the animal, the bitten part should be speedily removed by the knife or caustic, or both, and the wound kept open some time. In the case related in our last number, which occurred in Rose Street, Soho, and the accuracy of which account I ascertained by personal inquiry, these precautions were negleted. In a case of another female child, about 6 years old, whom I saw this day in the Middlesex hospital, the same neglect both of the dog and the wound took place; and the consequence was alike fatal; but in this last case, the wound being very slight, the symptoms did not appear till about four months after the bite, and the disease itself was much slower in its progress; for the patient lived six days after the inability of swallowing cold water was observed.

The errors which I wish more particularly to eradicate are, the belief that human beings, and other animals besides those of the dog and cat kind, can inoculate this erethism by their bite. No fact in favour of such an opinion has ever been observed. This mistaken notion has given birth to others; such as, that human beings in the late stages of the disease, rave, bark like dogs, and endeavour to bite the by-standers. This is never the case, unless the patient is outraged by brutal treatment, and then the same conduct would be exhibited in any disease.

Connected with these false opinions is the very general belief that the laws permit, at least, if not command, medical attendants to put a period to the sufferings of the patient, and the fears of relations, by smothering or bleeding to death. I take it for granted, that no practitioner would incur the penalty of felony without benefit of clergy, by adopting such a practice. The idle custom of sending persons and other animals wounded by the bite of of a rabid dog, to be dipped in the sea, is become nearly obsolete, in consequence of the repeated proofs of its inefficacy.

Having endeavoured to point out and remove these more dangerous and important errors, I shall state very briefly what is known and wanting to be known respecting rabid erethism. It would have been a most desirable circumstance for medical men to have been able, from the appearance of the bitten part, to have decided that the dog was rabid or otherwise; this is not the case, and the wound, unfortunately, heals quite as readily as any other of the same extent.

The period which elapses between the date of the bite and the appearance of the symptoms is very uncertain: the most usual is from five to seven weeks; and if symptoms similar to hydrophobia appear in less than three weeks, some other disease, particularly tetanus, which is nearly allied to rapid erethism, and often mistaken for it, ought to be suspected. The case in Rose Street, exhibited decided symptoms on the 27th day after the bite; but here the injury was extensive, and the quantity of poison applied to the wound was great. The reverse of all this took place in the case I saw in the Middlesex Hospital; but as I understand the physician under whose care that patient was admitted, intends to publish a detailed account of it, I shall not attempt to anticipate any of his observations. In the case of the Honourable Mrs. Duff, the disease occurred a year after the very slight wound that was inflicted.

Whatever be the interval, the first, and always most predominant symptom, through the whole course of this rapid disease, is an inability to bear the touch of cold water on any part of the body, and hence the name hydrophobia, or dread of water, came to denote the whole erethism. An attentive observer, however, may detect a lowness, a dejected look, and derangement of function, one or two days before the disease is confirmed; but this is seldom attended to, as being supposed to arise from some other cause. When the symptoms are established, cold air brings on the same spasms as cold water, and these spasms rise to convulsions, which increase in violence and frequency, till the uniform termination of the disease in death, releases the patient.

It may not be improper to mention, very briefly, what has been done, with a view to alleviate or cure this hitherto incurable malady. As the spasmodic symptoms always appear appear most urgent and distressing, blisters about the neck, warm bathing, opiates internally, mercurial friction, friction with olive oil, have all been exhibited with diligence and perseverance, but without the least advantage. It has indeed been long observed, that antispasmodics, both in tetanus and this erethism, totally fail of producing their usual effects.

I may perhaps be expected to suggest some new mode of treatment, but this is not to be obtained without much experience, which can scarcely fall to the lot of any individual in the present state of society. If the legislature of the country would sanction a conservatory for rabid animals, similar to that built by the Earl of Stamford for the preservation of foxes, such experiments might be instituted as would, doubtless, contribute essentially to the developement of the nature and laws of action of this most pernicious and subtil poison. If this however should be thought of too little importance to engage the attention of government, there can be no impropriety, that I can see, in prohibiting any dogs from being at large in the streets of towns and villages, where they can do no good, and may do incalculable mischief.

Some diseases, like some words, disappear, and others spring up in their stead. We know that leprosy disappeared about the time that *lues* was first seen in Europe. The small-pox is disappearing, and hydrophobia is becoming epidemic. Scarcely a month passes without instances of it in the metropolis. If any of our readers should not be disposed to place reliance on my opinions, and still contend that there is no such fatal disease as I have mentioned, I would refer them to the case of Mr. Bellamy of Holborn Hill, published by Dr. Fothergill; the case attended by Dr. Blackbourn, at Kensington; another by Mr. Ford of Golden Square; that published by Dr. Powel, which occurred so lately in St. Bartholomew's Hospital; that in the Middlesex Hospital; and that in Rose Street. And if these are not sufficient, I would then recommend them to peruse the elaborate Treatise on Hydrophobia by Doctor Hamilton.

*Pall-Mall Court, Nov. 16.*

CRITICAL

