# Corrigenda

**Published:** 1808-12

**Authors:** 


					jUne J1 from the bottorn, page 544, for never, read fcarccly ever,
END OF VOL. XX,

				

